# Identifying and Characterizing Medical Advice-Seekers on a Social Media Forum for Buprenorphine Use

**DOI:** 10.3390/ijerph19106281

**Published:** 2022-05-22

**Authors:** Gian-Gabriel P. Garcia, Ramin Dehghanpoor, Erin J. Stringfellow, Marichi Gupta, Jillian Rochelle, Elizabeth Mason, Toyya A. Pujol, Mohammad S. Jalali

**Affiliations:** 1 H. Milton Stewart School of Industrial and Systems Engineering, Georgia Institute of Technology, Atlanta, GA 30332, USA; giangarcia@gatech.edu; 2 Computer Science Department, University of Massachusetts Boston, Boston, MA 02125, USA; ramin.dehghanpoor001@umb.edu; 3 Harvard Medical School, MGH Institute for Technology Assessment, Boston, MA 02115, USA; estringfellow@mgh.harvard.edu (E.J.S.); marichi.gupta@gmail.com (M.G.); jillianrochelle24@gmail.com (J.R.); emason2@wellesley.edu (E.M.); 4 RAND Corporation, Arlington, VA 22202, USA; tpujolm@rand.org; 5 Sloan School of Management, Massachusetts Institute of Technology, Cambridge, MA 02139, USA

**Keywords:** opioid use disorder, buprenorphine-naloxone, Suboxone, advice-seeking, social network analysis

## Abstract

Background: Online communities such as Reddit can provide social support for those recovering from opioid use disorder. However, it is unclear whether and how advice-seekers differ from other users. Our research addresses this gap by identifying key characteristics of r/suboxone users that predict advice-seeking behavior. Objective: The objective of this analysis is to identify and describe advice-seekers on Reddit for buprenorphine-naloxone use using text annotation, social network analysis, and statistical modeling techniques. Methods: We collected 5258 posts and their comments from Reddit between 2014 and 2019. Among 202 posts which met our inclusion criteria, we annotated each post to determine which were advice-seeking (*n* = 137) or not advice-seeking (*n* = 65). We also annotated each posting user’s buprenorphine-naloxone use status (current versus formerly taking and, if currently taking, whether inducting or tapering versus other stages) and quantified their connectedness using social network analysis. To analyze the relationship between Reddit users’ advice-seeking and their social connectivity and medication use status, we constructed four models which varied in their inclusion of explanatory variables for social connectedness and buprenorphine use status. Results: The stepwise model containing “total degree” (*p* = 0.002), “using: inducting/tapering” (*p* < 0.001), and “using: other” (*p* = 0.01) outperformed all other models. Reddit users with fewer connections and who are currently using buprenorphine-naloxone are more likely to seek advice than those who are well-connected and no longer using the medication, respectively. Importantly, advice-seeking behavior is most accurately predicted using a combination of network characteristics and medication use status, rather than either factor alone. Conclusions: Our findings provide insights for the clinical care of people recovering from opioid use disorder and the nature of online medical advice-seeking overall. Clinicians should be especially attentive (e.g., through frequent follow-up) to patients who are inducting or tapering buprenorphine-naloxone or signal limited social support.

## 1. Introduction

Social media platforms such as Twitter, Facebook, and Reddit have fostered communities that provide solidarity and support for people dealing with a multitude of issues such as eating disorder [1], suicidal ideation [2], and chronic illnesses such as rheumatoid arthritis, stroke [3], HIV/AIDS [4], and opioid use disorder (OUD) [5]. In these communities, users receive emotional support, information, and companionship while, in some cases, preserving anonymity. Past research has described how social media platforms are used to benefit (or, in some cases, harm) subpopulations with certain medical conditions. For instance, social media can allow harmful or misleading opinions to propagate to other users [1,2], or offer an avenue for subpopulations to seek support, advice, or information relevant to their condition [3,4,5]. Studies exploring discussion on stroke- and HIV-specific forums have shown that participants largely use such forums to request and share information or emotional support, or to share their own experiences [3,4].

Research has provided some insight into online communities for people who use opioids, where advice-seekers and advice-givers often congregate. The anonymity of online forums such as Reddit has the potential to reduce stigma and social exclusion associated with illicit opioid use and can be an important factor for seeking support online [6,7]. These investigations have typically helped understand various stages of opioid use and misuse, for instance, to predict the transition to OUD [8] or determine the prevalence of polydrug use [9]. A separate body of research has provided insight on the social structure of these communities finding that, similar to in-person support groups such as Alcoholics Anonymous and Narcotics Anonymous (AA/NA) [10], community cohesion is driven by a core of long-standing members [11] while those most engaged with the platform (i.e., posting most frequently) are currently withdrawing from or still using illicit opioids [12]. More recently, an analysis of Reddit forums found that there is also an abundance of medical advice from non-clinicians including unverified OUD treatment alternatives [5,13]. Buprenorphine-naloxone is one of the most effective tools for reducing overdoses [14,15]. Given that users of this medication may seek support online, it is critical to characterize those seeking advice, in terms of their buprenorphine use status and their social connectedness to others, so that the public health response can adequately respond to inaccurate information while promoting evidence-based information. In the context of a complex and unrelenting opioid overdoses crisis [16,17], it is essential that all venues with which people who use opioids interact provide sound, evidence-based advice.

To address this research gap, we identify the characteristics of people who seek medical advice for OUD recovery on Reddit. Specifically, we analyze user and social network attributes of r/suboxone, a community on Reddit with a focus on discussions related to Suboxone^®^, a brand name for buprenorphine-naloxone. Suboxone is the most commonly discussed brand on Reddit and an effective medication used to support reduced use of opioids, and thus reducing overdoses [14]. Both Suboxone and buprenorphine use, including the mono-formulation of buprenorphine, without naloxone, are discussed in this forum, and we use the terms interchangeably. We combine text annotation, social network analysis, and statistical analysis to quantify the relationship between advice-seeking, buprenorphine-naloxone use, and social connectedness within this online community. The techniques described here are generally applicable to discussion on other online forums and social media beyond r/suboxone; we apply them here to improve the understanding of those who seek buprenorphine(-naloxone)-related advice from online platforms.

## 2. Materials and Methods

### 2.1. Data Description

Our data consisted of posts and comments collected from the “r/suboxone” subreddit, a sub-community of Reddit described as “a community for all things buprenorphine.” A labeled snapshot of the r/suboxone homepage and an example post and its comments are shown in Figures S1 and S2 in Multimedia Supplementary S1. We collected data from r/suboxone spanning 4 February 2014 (the inception of this subreddit) to 31 December 2019, excluding content created after 1 January 2020, to mitigate the potential effects of the COVID-19 pandemic on Reddit users’ posting behavior. We used the pushshift.io API [18] to collect URLs from all posts in this time period and used RedditExtractoR [19] library in R to extract relevant data (see Table S1 in Multimedia Supplementary S2) and subsequent comments from each post.

### 2.2. Exclusion Criteria and Data Sampling

To extract the most relevant posts for our analysis, we first excluded empty and deleted posts since no text can be extracted from them, and network characteristics cannot be computed for users with deleted accounts. We then excluded all posts made by authors without one prior post, since users with no prior post-activity would have no standing connections to other users and thus no network characteristics to analyze. Finally, because we were interested in medical advice-seeking, we narrowed our study sample to posts mentioning specific doctor/provider-related or buprenorphine- and Suboxone-related keywords (see Table S2 in Multimedia Supplementary S2), and the comments associated with these posts.

### 2.3. Annotating Advice-Seeking Posts and Buprenorphine Use Status

With the final study sample, each post was annotated as advice-seeking or non-advice-seeking, in addition to the estimated status of the poster’s current buprenorphine use. Three bachelor’s level research assistants [MG, EM, JR] and one Ph.D. student [RD] with backgrounds in medical informatics (all supervised by a substance use services researcher with expertise in qualitative coding and analysis [ES]) each annotated an initial 10 posts and collaboratively defined the criteria for the advice-seeking and buprenorphine use status criteria. A post was designated as “advice-seeking” if the user asked a specific question in their post about addiction, buprenorphine, or doctor-related issues. Examples of posts annotated as advice-seeking and not advice-seeking are shown in Table S3 in Multimedia Supplementary S2.

For annotating the buprenorphine use status, three categories were considered: “using buprenorphine,” “used to be on buprenorphine,” and “cannot discern.” A user was annotated as “using buprenorphine” if the content of their post indicated they were actively using Suboxone or its generic forms. Users who were identified as “using buprenorphine” were further classified as “inducting,” “tapering,” or “other.” A user was annotated as “inducting” if they described just beginning or being about to begin buprenorphine treatment, “tapering” if they described decreasing their dosage of buprenorphine with the intention to stop taking buprenorphine, and “other” if they described neither inducting nor tapering. We combined inducting and tapering into a single “inducting or tapering” category since both categories comprise transition stages. Users were annotated as “used to be on buprenorphine” if they mentioned past use of buprenorphine but indicated they have since stopped the treatment, or “cannot discern” if they did not give enough details to discern their buprenorphine use status. Uncertainties regarding annotations for specific posts were discussed and deliberated. Examples of posts annotated by buprenorphine use status are shown in Table S4 in Multimedia Supplementary S2.

### 2.4. Measuring Social Connectedness

To characterize social connectedness, we constructed a social network graph for each sampled post based on a timeframe defined by the posting user’s first post or comment on r/suboxone and ended on the date at which they made the sampled post. All posts and comments made outside of this time period were not considered.

To construct each post-defined social network graph, we modeled nodes as unique users and edges as relations between two users. We added a directed edge, i.e., a relation, from user A to user B if either: (1) user A created a post and user B commented on that post, or (2) user A commented on a post and user B replied to that comment. The weight of each edge is equal to the number of relations between the two users on r/suboxone. We illustrate this process in Figure S3 in Multimedia Supplementary S1.

For each posting user, we computed their life span, total degree, eigencentrality, closeness, authority score, and hub score based on the user’s network at the time they made their post. A user’s lifespan is the total number of days between their first post or comment on r/suboxone and the date at which they authored the sampled post. A user’s total degree is the total number of relations to and from that user. Eigencentrality measures how influential a node is within the network [20]. For example, a user who is connected to many “important” users (i.e., other users with high eigencentrality) will have a relatively high eigencentrality. Closeness is equal to the inverse of the average length of the shortest paths to/from all the other vertices in the graph [21]. In other words, a user who is “close” to all other users in the social network (e.g., through direct connections with all other users or having direct connections with users who have many direct connections to all other users) would have a high closeness score. Finally, a user’s authority score and hub score represent two related centrality measures [22]. In this context, users with high authority scores will tend to receive comments from other users who frequently reply to others’ posts. Likewise, the users who tend to reply to others’ posts will have high hub scores.

### 2.5. Statistical Analysis

We computed the total number of posts as well as the mean, standard deviation (SD), median, and inter-quartile range (IQR) for all network characteristics and the number and proportions of posts by buprenorphine use status. We then divided our data into advice-seeking and not advice-seeking posts and repeated this analysis. Differences between advice-seeking posts and not advice-seeking posts were analyzed using the Mann–Whitney U test for all numerical study variables and the Pearson’s Chi-squared test for the buprenorphine use status variables. To determine which (estimated) specific buprenorphine use statuses were driving significant differences in buprenorphine use status between advice-seekers and non-advice-seekers, we also conducted a post-hoc Chi-squared analysis on expected residuals [23] using the Benjamini–Hochberg *p*-value correction for multiple comparisons [24].

To quantify the relationship between advice-seeking (vs. not advice-seeking) with a user’s social connectivity and buprenorphine use status, we constructed four generalized linear models (GLMs) with logit link functions. In each model, the dependent variable is given by a binary variable representing whether a post is advice-seeking or not. The independent variables include the posting user’s network characteristics and buprenorphine use status, the latter being re-coded as a series of binary variables using one-hot encoding with “used to be on buprenorphine” as the reference category. These linear models are used only to identify advice-seeking status, and are not intended to be used as clinical decision support tools.

The *stepwise model* aimed to identify a parsimonious set of independent variables through statistical variable selection (i.e., using forward-backward selection). We compared the stepwise model to three additional models: the full model, buprenorphine use model, and network model. The *full model* contained all network characteristics and the buprenorphine use status variables. The buprenorphine *use model* and *network model* contained only the buprenorphine use status and network characteristics variables, respectively. To aid our inference of modeling coefficients, we computed the variable inflation factor to assess the multicollinearity of modeling variables for each model. We then applied leave-one-out cross-validation to evaluate each model using area under the receiving operator characteristic curve (AUROC), Akaike information criterion (AIC), and F1 score. Altogether, these measures provide a holistic picture of each model’s predictive performance.

## 3. Results

### 3.1. Data Characteristics

**Figure 1:**
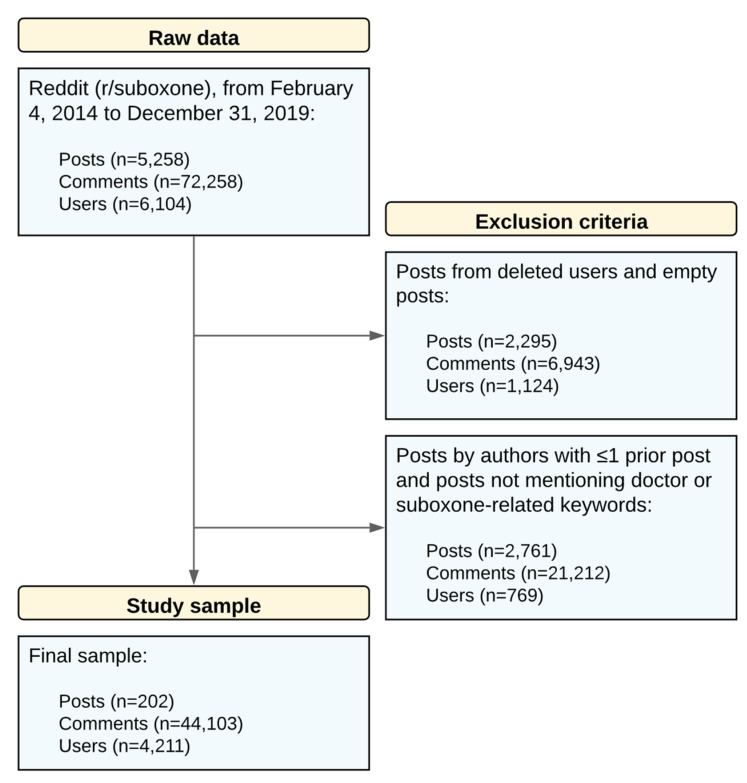
Application of exclusion criteria to raw data for obtaining the study sample.

The final study sample contained 202 posts (see Figure 1). Table 1 summarizes these data with respect to our study variables. Within these posts, 137 (67.8%) were advice-seeking.

Among posting users, those who made advice-seeking posts had a significantly different total degree (*p* = 0.004), eigencentrality (*p* = 0.003), authority score (*p* = 0.014), and hub score (*p* = 0.007) than users who did not make advice-seeking posts. Additionally, the proportion of users in each buprenorphine use status was significantly different between users who authored advice-seeking posts and those who did not (*p* < 0.001). Notably, there was a significantly greater proportion of advice-seeking users vs. not advice-seeking users who were inducting or tapering (*n* = 57, 41.6% vs. *n* = 14, 21.5%; *p* = 0.02) and a significantly lesser proportion of users who used to be on buprenorphine (*n* = 6, 4.4% vs. *n* = 14, 21.5%; *p* = 0.001).

Sample posts representative of the annotation categories are available in Multimedia Supplementary S2 (Table S3) and exhibit the discussion content on the forum. These posts indicate that advice-seekers request information on topics such as symptom management, whether under changing or stable dosages, or about how to broach conversation topics/navigate disagreements with their provider. For instance, one advice-seeking user writes *“[My doctor] wants to taper me down from 8 mg to .25 in two months. Then put me back on oxycodone for one month and taper from that… Isn’t this idea sort of crazy? Giving me opiates again?”* Non-advice-seeking posts, on the other hand, may instead offer general experience or guidance to others, or share their personal worries and struggles.

Figure S4 in Multimedia Supplementary S1 contains further examples of advice-seeking and not advice-seeking posts, along with the posting user’s social network graph, post characteristics, network characteristics, and buprenorphine use status. These examples were selected to be close to the mean total degree in each category. The advice-seeking post shows a user whose post includes the text “... *I just need help from you guys... Please help me taper with a plan*...”. As such, we labeled this user as “Using buprenorphine: Tapering.” Based on this user’s position in the social network, they have a total degree of 48, closeness of –0.24, eigencentrality of 0.11, authority score of 0.11, and hub score of 0.06. The date of this post was 287 days after the user’s first post or comment or comment on r/suboxone, leading to a lifespan of 287 days. For comparison, the not advice-seeking post example includes “*I finally got my dr to prescribe Subutex instead of suboxon … Much more of a clean feeling I guess. Hope it lasts.*” Hence, we labeled this user’s buprenorphine use status as “Using buprenorphine: Other.” This post was made 88 days after the user’s initial post or comment on r/suboxone, leading to a lifespan of 88 days. Moreover, the user’s social connectedness on r/suboxone was quantified with a total degree of 85, closeness of −0.23, eigencentrality of 0.32, authority score of 0.28, and hub score of 0.19. Notably, the advice-seeking user’s measures of social connectedness are all lower than the user who is not advice-seeking, with the exception of closeness.

### 3.2. Regression Modeling

Our GLMs are described in (Table 2). In the stepwise model, “total degree,” “closeness,” and the buprenorphine use status variables were selected by the variable selection procedure, with “total degree” (*p* = 0.002), “using buprenorphine: inducting/tapering” (*p* < 0.001), and “using buprenorphine: other” (*p* = 0.002) being significantly different from 0 (i.e., strongly associated with advice-seeking). These three variables had variance inflation factors (VIF) ranging from 1.04–1.08, indicating low multicollinearity, and none of the variables that were removed by the stepwise variable selection procedure were significant in any of the other GLMs. Additionally, all variables that were significant in the stepwise model were significant in at least one other GLM. Notably, VIFs in all other models were low-moderate (i.e., VIF ≤ 5) except for eigencentrality (VIF = 10.94–11.26) and authority score (VIF = 9.53–9.69) within the full and network models. Nevertheless, these variables had coefficient estimates close to 0 and were not significant in either model.

Whether each variable increased/decreased the likelihood of being an advice-seeker (i.e., whether the coefficient was positive/negative) was consistent across all models. Among variables with coefficients significantly different from 0, “total degree” had a negative coefficient, indicating that posting users with more connections were less likely to be advice-seeking. Likewise, the coefficients for “using buprenorphine: inducting/tapering” and “using buprenorphine: other” were positive, indicating that posting users who were identified as using buprenorphine were more likely to be advice-seeking than users who were identified as formerly using buprenorphine.

With regard to performance measures, the stepwise model outperformed all other models with the greatest AUROC (0.66 vs. 0.52–0.61), least AIC (231.86 vs. 239.19–246.85), and greatest F1 score (0.47 vs. 0.30–0.44).

## 4. Discussion

Our analysis focused on identifying characteristics of advice-seekers in a sub-Reddit online forum (‘r/suboxone’), wherein the use of buprenorphine is discussed among people who use opioids illicitly. Buprenorphine and buprenorphine-naloxone are effective treatments for OUD [14,25,26], but online communities can provide potentially risky non-expert medical advice [5]. It is important to identify characteristics of people who might similarly be seeking non-expert medical advice regarding their use of buprenorphine.

At the intersection of OUD and social media, previous studies have separately analyzed social roles across various subreddits [7], advice-seeking among users with OUD [27], and posts regarding inducting and tapering [28,29]. Commonly, these studies have found that advice-seeking and advice-giving, especially with regard to managing OUD, play an important role on these social media platforms. Although our analysis did not focus on the content of the posts, we found that the nature of discussion and advice-seeking is similar to findings in these previous works, as well as online support forums for stroke and HIV/AIDS [3,4]. Specifically, we find that most posts in our annotated sample are advice-seeking, requesting information or guidance on buprenorphine-specific topics. Inspection of sample posts reveals that such posts ask about topics including symptom management and doctor interactions. Online user questions about how best to interact with or challenge their providers are indicative of a lack of trust with medical professionals, which has been documented as common among those with OUD [30,31]. In addition, the stigma around OUD may cause providers to discriminate against those seeking medical treatment, potentially increasing mistrust between medical professionals and people who use opioids [32,33]. Therefore, a lack of comfort in discussing concerns with providers may be a potential driver for users to ask questions on this online forum.

In our best-performing model (i.e., stepwise), advice-seeking was associated with people who are inducting onto or tapering off of buprenorphine and with having fewer (i.e., total degree) and less close (i.e., closeness) social connections, while other measures of social network status provided no additional information. This mirrors similar dynamics to those in 12-step groups like Narcotics Anonymous (NA), where new members are encouraged to seek advice and support from more experienced sponsors [10]. Similarly, other research has found people feel more comfortable seeking advice from their peers than from providers [34]. While we did not assess their comfort level with providers, the fact that people are turning at all to non-expert peers for medical advice-not just support-is important insight given these transitional stages are risky (e.g., for overdose), and can be critical to success [26].

In contrast, Reddit users with more connections who no longer use buprenorphine were present, but less likely to seek advice, similar to in-person recovery support communities like Narcotics Anonymous [10]. We did not investigate their role—which often appeared to be as advice-givers-but they might best be used in connection with health mediators, expert patients, or other health professionals online who can help safely guide users in transition stages. Such supportive, trained teams could focus specifically on dealing with stress, which is associated with advice-seeking among people in recovery [35], and maybe especially common among people who are initiating or tapering buprenorphine, partly due to intense withdrawal symptoms [36,37].

There is still a clear role for prescribing clinicians, who should pay special attention (e.g., by providing more frequent follow-up and being especially accessible) to people who are inducting or tapering buprenorphine. However, recommendations generally focus on dosing levels or how to identify when it is appropriate to begin inducting or tapering with buprenorphine [26,38,39]. As clinical best practices and public health interventions for OUD treatment continue to evolve, it will be critical to understand why people in transition stages turn to online platforms, what specific advice they are seeking, and how their medical questions could be better addressed by providers.

Beyond the clinic, quickly and accurately identifying advice-seeking users can help online platforms automate the delivery of medically sound informational resources (e.g., via chatbots [40]) for people recovering from OUD. Since our stepwise model achieved greater predictive accuracy than the network and buprenorphine use models, these results suggest that the combination of network characteristics and buprenorphine use status are better indicators of advice-seeking behavior than either of those factors individually. Notably, the network model attempted to include a more comprehensive description of each user’s network compared to the stepwise model. However, none of the additional variables beyond the total degree were significant, and the network model had far worse predictive performance than the stepwise model. These findings indicate the importance of focusing on the right measures of social connectedness when attempting to identify advice-seekers on online platforms. Fortunately, the total degree is relatively simple to compute. Hence, if buprenorphine use status can be classified with relatively high accuracy (e.g., using natural language processing methods), then our stepwise model can provide a starting place for identifying users who might benefit from targeted medically sound advice on online platforms. Research into whether users of online platforms would welcome such advice is warranted.

This research is not without its limitations. First, this study focuses only on advice-seeking on r/suboxone, which narrowed the sample size to only 202 posts. Future research can consider additional social roles, including users who give advice or social support, on additional opioid-related subreddits such as r/opiates, which would explore a greater volume of posts as well as other treatment modalities. Second, our study focused on the characteristics of advice-seeking users and not the characteristics of the posts themselves. Additional insights can be drawn from analyzing the content of the posts to highlight patterns of advice-seeking posts and facilitate the automated identification of advice-seekers. This analysis could even be extended to evaluate the quality of advice shared on these online platforms using qualitative analysis methods. Third, our work leveraged manual annotations of users’ posts to determine respective buprenorphine use statuses. In particular, the manual classification of the user’s stage of buprenorphine use, while systematic, was subjective. Further, our analysis was limited to the information in each user’s post. Future research may explore algorithmic techniques (e.g., [41]) to classify such users, which in tandem with the model presented here, would streamline the identification of advice-seeking users and facilitate analysis of topics for which advice is often sought. Finally, our study is limited to data ending on 31 December 2019. The onset of COVID-19 has brought many challenges to PWUO, which could have changed the nature of their online activity and interactions.

## 5. Conclusions

In this research, we offer a general methodology to identify medical advice-seeking users on social media based on analyzing the network characteristics and post-content of users. We implemented our approach to assess the relationship between advice-seeking, social connectedness, and buprenorphine-naloxone use status on Reddit. While previous studies have analyzed social roles and connectedness of subreddits [7], advice-seeking among users with OUD [27], and posts regarding induction and tapering [28,29], our study is the first to connect the three topics and to do so by combining social network analysis, text annotation, and statistical modeling. Here we have (1) demonstrated a method to classify advice-seeking users based on their network characteristics and buprenorphine-naloxone use stage; (2) shed light on the characteristics of advice-seekers on an online platform for OUD recovery; and (3) provided insights for the clinical management of PWUO who are recovering from OUD as well as the nature of online medical advice-seeking. Given the vulnerability of PWUO, it is imperative that future research continues to explore the needs of this population and how they can be met. Further, the techniques used here can be used on other forums and social media discussing other health topics.

## Figures and Tables

**Table 1:** Description of data with respect to post characteristics, network characteristics, and buprenorphine use status.

	Variable	Full Study Sample	Advice-Seeking	Not Advice-Seeking	*p*-Value ^a^
	Total number of posts ^b (%)^	202 (100%)	137 (67.8%)	65 (32.2%)	
		**Mean (SD)** **Median (IQR)**	**Mean (SD)** **Median (IQR)**	**Mean (SD)** **Median (IQR)**	
**Post characteristics**					
	Length of post (words)	297 (390.7) 165.5 (227.5)	214 (222.9) 145 (127)	472 (572.8) 278 (482)	<0.001
	Comments per post	12.9 (14.1) 8 (19.75)	11.7 (11.7) 9 (19)	15.3 (17.9) 8 (18)	0.529
**Network characteristics**					
	Total degree	44.5 (54.5) 26 (38.25)	33.9 (32.1) 24 (32)	66.6 (80.0) 35 (52)	0.004
	Closeness ^c^	0.0 (1.0) −0.22 (0.07)	−0.1 (0.7) −0.22 (0.08)	0.1 (1.4) −0.22 (0.06)	0.904
	Eigencentrality	0.2 (0.2) 0.12 (0.16)	0.1 (0.1) 0.11 (0.12)	0.2 (0.2) 0.18 (0.23)	0.003
	Lifespan (days) ^d^	178.8 (289.5) 59.83 (208.47)	159.7 (247.6) 63.83 (202)	219.1 (361.1) 48.83 (218)	0.848
	Authority score	0.2 (0.2) 0.12 (0.17)	0.1 (0.1) 0.11 (0.13)	0.2 (0.2) 0.16 (0.26)	0.014
	Hub score	0.1 (0.1) 0.05 (0.1)	0.1 (0.1) 0.05 (0.08)	0.2 (0.2) 0.09 (0.15)	0.007
**Buprenorphine use status**		***n* (%)**	***n* (%)**	***n* (%)**	<0.001 ^e^
	Using buprenorphine: inducting/tapering	71 (35.1)	57 (41.6)	14 (21.5)	0.02
	Using buprenorphine: other	102 (50.5)	70 (51.1)	32 (49.2)	>0.999
	Used to be on buprenorphine	20 (9.9)	6 (4.4)	14 (21.5)	0.001
	Cannot discern	9 (4.5)	4 (2.9)	5 (7.7)	0.33

^a^*p*-value compare the difference across advice-seeking and not advice-seeking posts. ^b^ Total number of posts excludes repeat posts by the same author. ^c^ Normalized values presented due to scale of variable. ^d^ Denotes lifespan of posting user. ^e^ *p*-value computed using Pearson’s Chi-square test to compare distribution across buprenorphine use status categories for advice-seeking and not advice-seeking.

**Table 2:** Model coefficients and performance measures for each model predicting the likelihood of advice-seeking.

	Model	Full ^a^	Network ^a^	Buprenorphinee Use ^a^	Stepwise ^a^
**Coefficient (95% CI)**					
	Intercept	−0.86 (−2.07, 0.24)	0.67 * (0.08, 1.25)	−0.85 (−1.89, 0.07)	−0.69 (−1.76, 0.28)
**Network characteristics**	Total degree	−0.82 (−1.70, −0.01)	−0.81 * (−1.65, −0.06)		−0.63 ** (−1.07, −0.25)
	Closeness	−0.33 (−0.83, 0.04)	−0.30 (−0.74, 0.05)		−0.31 (−0.69, −0.01)
	Eigencentrality	0.00 ^e^ (−1.18, 1.20)	−0.02 ^e^ (−1.20, 1.12)		
	Lifespan	0.00 (0.00, 0.00)	0.00 (0.00, 0.00)		
	Authority score	−0.05 ^e^ (−1.15, 1.07)	−0.02 ^e^ (−1.13, 1.09)		
	Hub score	1.69 (−2.77, 6.39)	1.18 (−3.19, 5.80)		
**Buprenorphine use status**	Using buprenorphine: inducting/tapering ^b^	2.10 *** (0.96, 3.34)		2.25 *** (1.17, 3.44)	2.08 *** (0.94, 3.31)
	Using buprenorphine: other ^b^	1.41 * (0.33, 2.59)		1.63 ** (0.63, 2.74)	1.42 * (0.35, 2.57)
	Cannot discern ^b^	1.02 (−0.83, 2.92)		0.62 (−1.04, 2.28)	1.09 (−0.67, 2.90)
**Performance Measures ^c^**					
	AUROC	0.61	0.54	0.52	0.66 ^d^
	AIC	239.19	246.85	241.02	231.86 ^d^
	F1	0.44	0.30	0.40	0.47 ^d^

* *p* < 0.05, ** *p* < 0.01, *** *p* < 0.001. ^a^ Full model contains all variables, Network model contains only network characteristics, Buprenorphine Use model contains only buprenorphine use status, and the Stepwise model contains independent variables determined by stepwise variable selection. ^b^ Buprenorphine use status variable binarized using one-hot encoding with “Used to be on buprenorphine” as the reference category. ^c^ AUROC: Area under the receiver operating characteristic curve; AIC: Akaike Information Criterion. ^d^ best-performing model. ^e^ variance inflation factor > 5.

## Data Availability

Publicly available datasets were analyzed in this study. This data can be found here: https://pushshift.io, https://www.reddit.com/r/suboxone.

## Supplementary Materials

The following supporting information can be downloaded at: https://www.mdpi.com/article/10.3390/ijerph19106281/s1; Multimedia Supplementary S1: Figure S1. Illustration and anatomy of the r/suboxone homepage and a post and its comments; Figure S2. Illustration and anatomy of a post and its comments; Figure S3. Illustration of social network graph construction; and Figure S4. Illustration of (A) advice-seeking and (B) not advice-seeking posts with posting user’s network; Multimedia Supplementary S2: Table S1. Features and descriptions of data collected from r\suboxone; Table S2. Doctor- and Suboxone-related keywords used to select posts for study; Table S3. Examples of advice-seeking and not advice-seeking posts; and Table S4. Examples of posts annotated by buprenorphine use status.

## Abbreviations

AA/NA	Alcoholics Anonymous and Narcotics Anonymous
AIC	Akaike Information Criterion
AUROC	Area Under the Receiving Operator Characteristic Curve
GLMs	Generalized Linear Models
MOUD	Medication for Opioid Use Disorder
OUD	Opioid Use Disorder
PWUO	People Who Use Opioids
SD	Standard Deviation
