# Supplementary material for: Identifying and Characterizing Medical Advice-Seekers on a Social Media Forum for Buprenorphine Use
Source: Int J Environ Res Public Health. 2022 May 22;19(10):6281. doi: 10.3390/ijerph19106281 (PMC9141384; doi:10.3390/ijerph19106281)
Supplement: Supplementary file 1 [file ijerph-19-06281-s001.zip › Multimedia Supplementary S1.pdf]

Multimedia Supplementary S1

Identifying and Characterizing Medical Advice-Seekers on a Social Media Forum for Buprenorphine Use

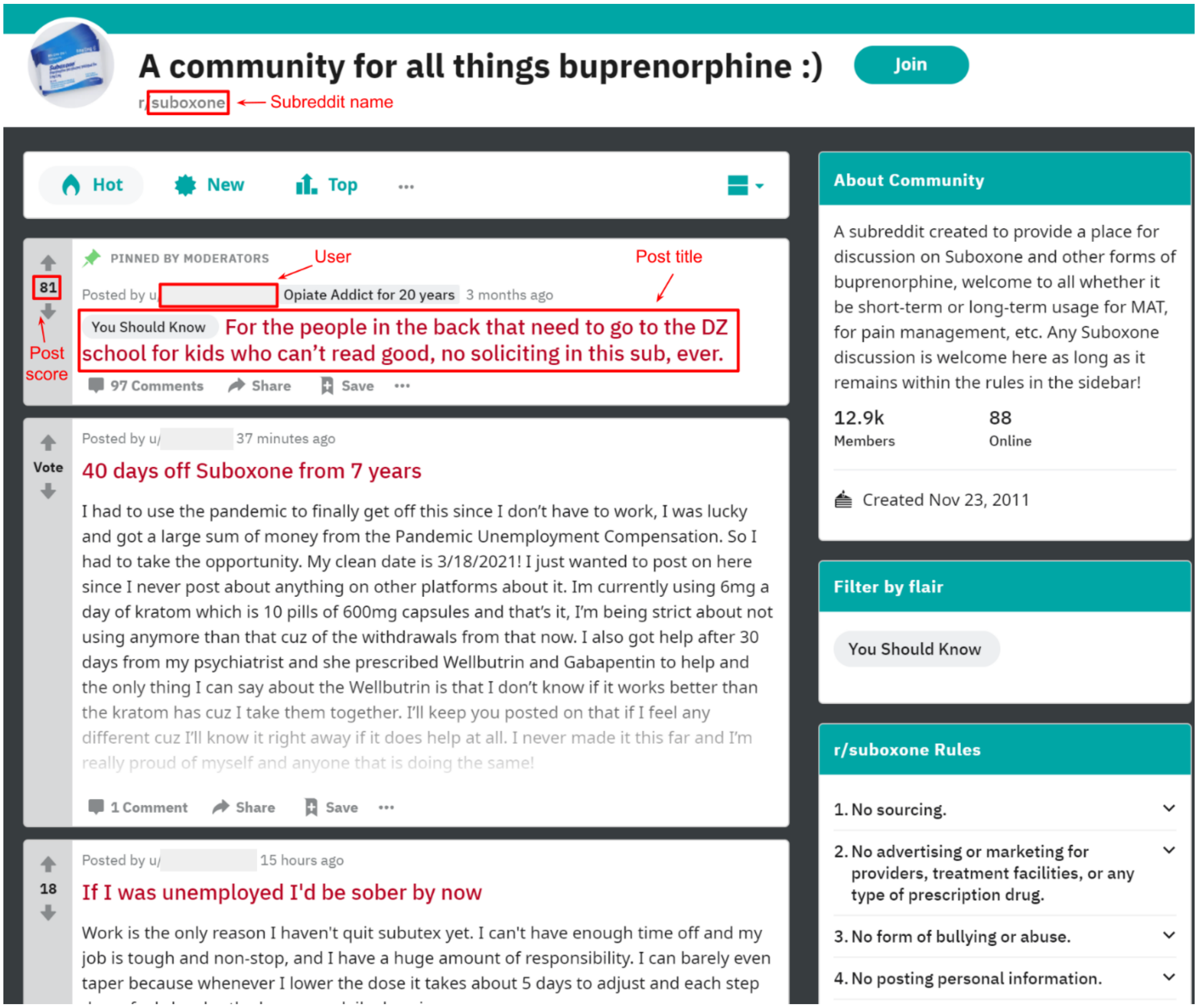

Figure S1: Illustration and anatomy of the r/suboxone homepage and a post and its comments

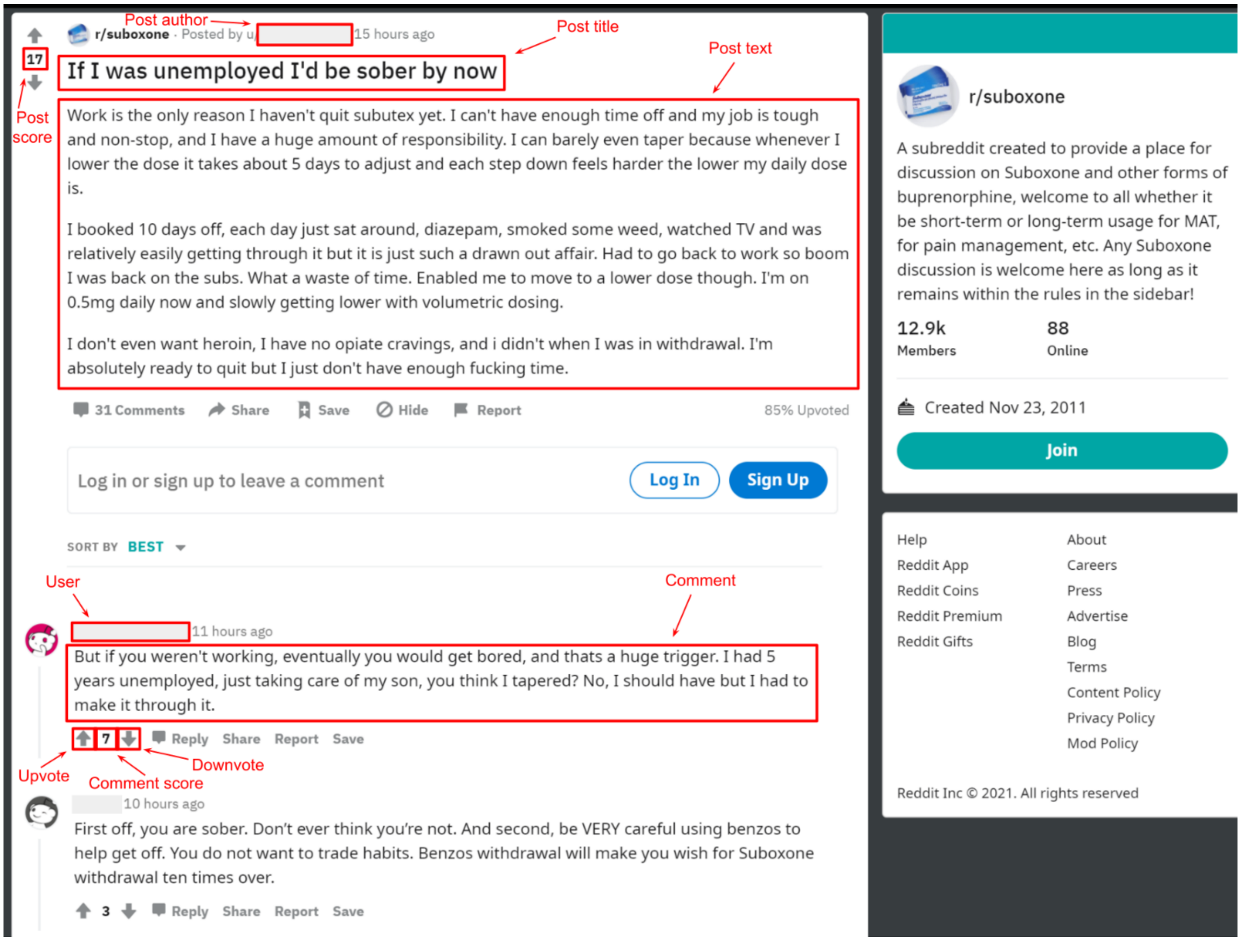

Figure S2: Illustration and anatomy of a post and its comments

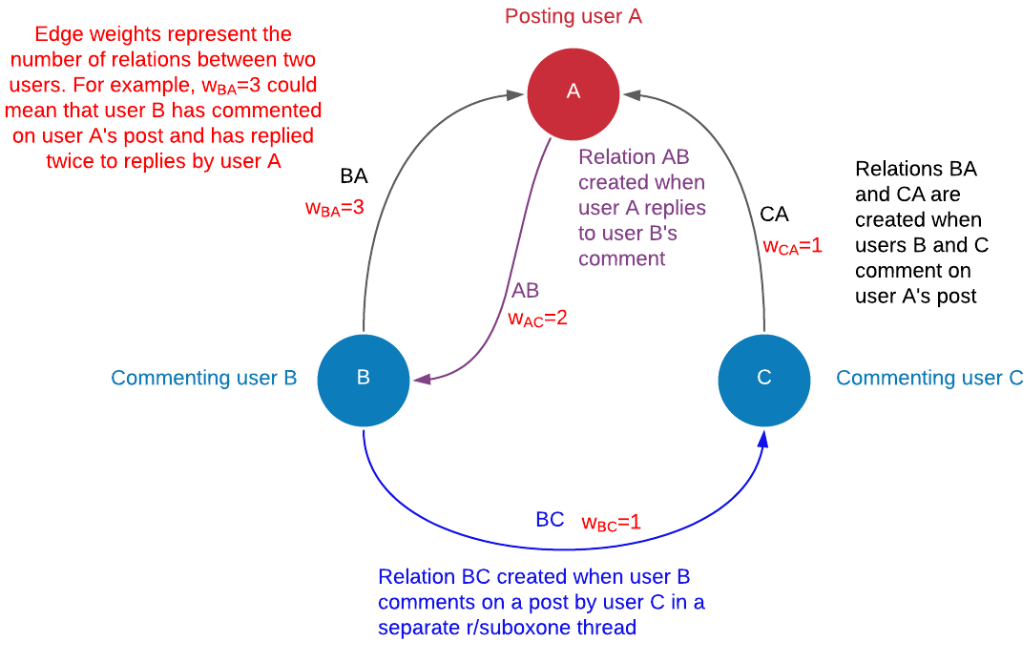

Figure S3: Illustration of social network graph construction

(A) Advice-seeking

Post title: “Need Support”

“Ok guys I purposely skipped my Sub doc appointment today . my reason , I am tired of taking this medicine and going to piss in a cup every month. Tired of looking like a junkie at the pharmacy. I am done!!! Now here's the thing, I do have 75 / 8 mg subs to taper down to zero!!! I am guessing that should be plenty. I just need help from you guys , you guys who understand me. Please help me taper with a plan . I have no idea the best way to do it. If anyone has time , I would be really greaful [sic]. thanks and I will keep this thread open and write down my progress .”

Characteristics:
Buprenorphine use status: Using buprenorphine: Tapering
Total degree: 48
Closeness[a]: -0.24
Eigencentrality: 0.11
Lifespan: 287 days
Authority score: 0.11
Hub score: 0.06

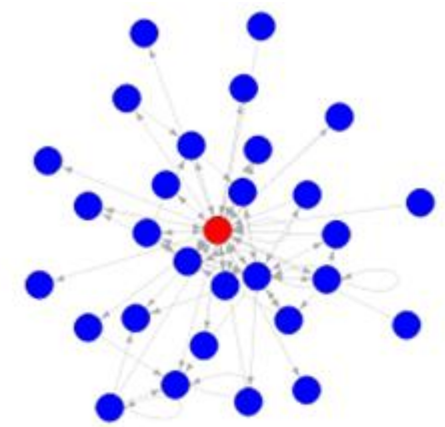

(B) Not advice-seeking

Post title: “Subutex”

“I finally got my dr to prescribe subutex instead of suboxon and I must say I don’t feel that gross anxiety feeling like suboxon gave me. Much more of a clean feeling I guess. Hope it lasts”
Buprenorphine use status: Using buprenorphine: Other
Total degree: 85
Closeness[a]: -0.23
Eigencentrality: 0.32
Lifespan: 88
Authority score: 0.28
Hub score: 0.19

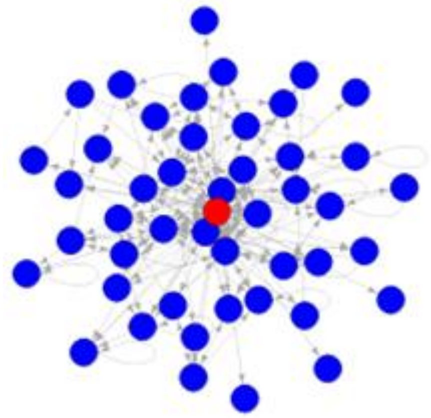

Figure S4: Illustration of (A) advice-seeking and (B) not advice-seeking posts with posting user’s network

Posting user shown as a red node; all other users shown as blue nodes; edges indicate the relation between users.
[a]Normalized value computed by subtracting the sample mean and dividing by the sample standard deviation
