# Supplementary material for: Identifying and Characterizing Medical Advice-Seekers on a Social Media Forum for Buprenorphine Use
Source: Int J Environ Res Public Health. 2022 May 22;19(10):6281. doi: 10.3390/ijerph19106281 (PMC9141384; doi:10.3390/ijerph19106281)
Supplement: Supplementary file 1 [file ijerph-19-06281-s001.zip › Multimedia Supplementary S2.pdf]

Multimedia Supplementary S2

Identifying and Characterizing Medical Advice-Seekers on a Social Media Forum for Buprenorphine Use

Table S1: Features and descriptions of data collected from r\suboxone

Feature	Description
Structure	Relates each comment or post to their reply comments
Post_date	Shows the dates each post created
Comm_date	Shows the dates each comment created
Num_comments	Number of comments for each post
Author	The author of each post
User	The author of each comment
Comment	The texts for each comment
Post_text	The texts for each post
Title	The post title
Lifespan[a]	Number of days between first post or comment and the current post or comment for each user

[a]Denotes a feature that was computed using collected data.

Table S2: Doctor- and Suboxone-related keywords used to select posts for study.

Doctor-related keywords	"doc", "docs", "doctor", "doctors", "dr", "drs", "gp", "general practitioner", "physician", "physicians", "primary care provider"
Suboxone-related keywords	"bupes", "buprenorphine", "suboxone", "subs", "soboxone", "dr reddy", "dr reddys", "bupe", "subutex", "naloxone", "bunavail", "bup", "bupenorphine", "buperenoprhine", "espanor", "norbuprenorphine", "sublocade", "subutext", "zubsolv"

Keyword lists were compiled by manually inspecting a list of all words appearing in the corpus to identify misspellings and abbreviations. Two doctor-related words were excluded due to possible multiple meanings: namely, “pcp” (Phencyclidine, or Angel Dust), was not included as an abbreviation for “primary care provider”, and “DOC” (all caps, drug of choice) was not included as an abbreviation for “doctor.” Care was taken to ignore the drug brand “Dr. Reddy” when searching for a doctor-related keyword.

Table S3: Examples of advice-seeking and not advice-seeking posts

	**Example 1**	**Example 2**
Advice Seeking	I just got the lab results back from some blood work I had done this past Monday because I told my doctor that I had basically no sex drive and that I always feel tired and lazy. I found out that my T level was at 150 when the average person has a testosterone level of between 300 and 800 I believe. I'm getting ready to find a endocrinologist and have all of my paperwork sent over there so that I can get started on the T injections for the time being to get my numbers up, but I have to run this by my Suboxone doctor first and foremost.<br><br>Right now I am on 16mg of testosterone and I've been on Suboxone for a year and a half. I've had some of the symptoms of low sex drive since before I started taking Suboxone but don't have a base line to compare it with, so we don't know for sure if the Suboxone is what is causing the low T count - but most of us who in an ORT (opioid replacement therapy) group know that painkillers, especially long-acting ones, can lower your T count quite a bit in some people.<br><br>I'm afraid that my doctor is going to want me to come down from 16mg of Suboxone daily and get me on a lower dose and I'm not prepared for that yet. I want to be on 16mg for at least until the end of summer and when it starts cooling off start stepping down. This is mainly because I sweat profusely even when it's not all that hot outside and find myself changing undershirts at least once a day because of the sweat even after using prescription antiperspirant to help with the sweats. When it gets cooler I will be more willing to start stepping down to 12mg and see how I handle that dosage. Does anyone think that my Suboxone doctor will want to lower my dosage levels, and will it matter that I'm receiving a schedule 3 substance from another doctor while I'm on Suboxone (another schedule 3 drug)? If my Sub doctor has to prescribe the testosterone I don't have a problem with that since all of my controlled substances would be scripted by the same doctor, but right now I'm afraid that if I have to start on weekly injections of T that he will want to lower my dosage to help with my T levels and I don't think I'm ready to step down for another 3-4 months or so at the least. If anybody out there is receiving Suboxone as well as some form of testosterone I would really love to hear from you and your experience regarding taking both substances together. Thanks for any input you might have regarding this situation.	Can anyone tell me their about experience tapering off of subs? Im on 8mg a day and have been for a long time but I really really want to be off of them. The nalaxone makes me feel like shit all the time and I get cold sweats. I want to start tapering after my next appointment with my doctor but Im really scared
Non-Advice-Seeking	I have taken my last dose of sub today so here we go! Wish me luck fam. Im really nervous and anxious but that is to be expected. I have been on subs for 3 months and 27 days. Started at 12mg a day and just jumped from .5 mg a day. I wanted to taper lower but lacked the self control it took, I would cheat on days sometimes taking 2-4 but for the most part over the last month Ive taken 0.5 to 1 mg a day. Im really worried what this is going to bring but Im hoping that all I am doing is building it up in my head. Before subs I had a 30-60 mg oral oxy habit, not crazy but enough to cause bad withdrawals. Im really ready to be done with all the dependent chemicals. I have gabapentin but I am not sure I will use that. Also have low dose benzos for if and when I cant sleep, Im sure that will come. I am completely out of subs and Im not going back to the sub doctor. Im also not going back to oxy. Im ready for a normal life and Ive put in the work to put myself in a position to be clean. Just scared of whats to come the next couple of weeks but I know it will pass. I am hoping that since I havent been on subs long that it wont be unbearable.<br><br>Thanks for listening >>	I feel like Dr.'s who prescribe buprenorphine don't have enough of an education on opioid addiction. Just because the only true education is from experience. They can't know what we know if they've never been there.<br>They don't know how strong buprenorphine is, and will have a hard time prescribing accurate & appropriate doses to patients.<br>They also don't know how an addicts mind works and the lack of self discipline & self control addicts have in relation to opioids.<br>They can't comprehend that their patients might have a hard time following their prescribed dosage. They don't understand the conundrum of prescribing the same class of addictive substance to treat a patient for their addiction to a similar substance.<br>Akin to using fire to put out a fire. Which does exist as controlled burning of flammable fauna to take away the fuel source for an approaching fire.<br>Which is a good analogy for how Suboxone should be used. The problem is that it's hard to do a controlled burn when you a Dr. are handing the tool to do so to someone with a lack of control in the first place.<br>Not saying that makes any of them bad doctors. Far from it. It's just that a short class or two that Dr.'s have to take to prescribe buprenorphine is so far from being enough info to really understand how complex addiction is.<br>The very first day I took Suboxone, I was given a full 8mg strip and was told to keep it under my tongue for 5 min. Then the Dr. Would observe me for 10 min. 10 minutes is not enough time for buprenorphine to fully kick in.<br>I had also been 2 weeks into withdrawal, and I had no idea how strong buprenorphine is. My tolerance was nil, and when it finally kicked in half an hour later I went from feeling like dog shit to superman.<br>Cut to 2 hours later, I started to get nauseous. I ended up puking 10 times that day. 8mg was way too much. Now that I'm experienced with Suboxone, I would have been just fine on 2mg strips. But I didn't ask my Dr. to put me on a lower dosage, or even tell him that I puked 10 times the first day. Why because I'm an addict, and I'll take more than less.

Table S4: Examples of posts annotated by buprenorphine use status

	**Example 1**	**Example 2**
Using buprenorphine: inducting	Additional question about starting subs tomorrow. I know i need do be in withdrawal and can't take benzos while on subs but can i take an ativan tonight for sleep since i won't be on oxys?<br><br>Update: at dr and the sub is currently dissolving. I really like the dr and he is NOT pushing a high dose. Wants me to take smallest possible to feel ok.	Hey, i start Suboxone on Monday because i just literally spend all day racking up lines of Oxy's and sniffing them, its all through a recovery program and stuff so its all good, i just wondered, i have no intention of abusing any drug or even drinking booze anymore, just taking my sub and getting on with my life, will my health improve from stopping the oxy and moving onto subs? like will my lungs stop hurting and my arms stop aching, will i stop waking up feeling like shit from wrecking my sinusus and all that bad stuff? sorry if this sounds really naieve to you guys but this is the first time ive ever asked for help for a problem and i now once i have this under control with the sub i wont be tempted to take anything else, im really into fitness and powerlifting outside of being a bit of a wreck head. So im hoping i can take it, get a bath get dressed and go to the gym get on with my life and feel better? anyway just a bit of a rant as im a bit anxious about seeing the doctor on Monday and aabout what will happen and stuff - nobody knows about my addiction but the recovery people, not even my folks who i live with.
Using buprenorphine: tapering	My new doctor wants me off subs within 2 months. I've been on them about 2 years. I just moved to another state and I think that's a great idea. However, it's the way he wants to do it. He wants to taper me down from 8mg to .25 in two months. Then put me back on oxycodone for one month and taper from that. He says the withdrawl from the oxy should be easier than coming off bupe. Isn't this idea sort of crazy? Giving me opiates again? He also seems to think if I take testosterone while I'm withdrawing from the percocets it will make it milder because it will give me energy. Has anyone heard of this? It just sounds bat shit crazy to me.	I made this account to make a post almost a week ago about how I couldnt stop taking Suboxone, even though it was causing issues in my relationship. I also mentioned that I was having stomach issues and was passing a concerning amount of blood when I went to the restroom.<br><br>I went to the doctor (not Suboxone doctor) to get checked out and to hopefully get some clonidine and something non-narcotic to help me sleep while tapering off. My doctor told me that I had ulcerative colitis in my stomach and an infection in my colon from the blood work results.<br><br>He asked me if I was still taking the Suboxone, I assume after he looked at my recent prescriptions. I confirmed that I was, but that I was trying to taper off currently and that I would not be going back to get another script. He said, point blank, Get off the Suboxone as quickly as you can. I believe your issues stem from taking this medication and its effect on the digestive system. I was taken back a bit, leaving with a script for just antibiotics and an appointment for a month later, but I reflected on it when I was alone.<br><br>Ive been going to NA recently, and Ive been trying to connect with my higher power. Ive been praying and meditating, anything I could to find what these people keep talking about. Ive literally been down on my knees begging for some sort of sign to help me move on from this for good. I dont want to take opiates anymore. Ive been putting some form of opiate into my body for the last 8 years. Ive wondered for a while how this is affecting my brain and my body, as its a long-term thing at this point, and I am afraid of doing irreversible damage to myself.
Using buprenorphine: other	So I went to the doc and was honest and everything worked out for the better... But now it feels like I'm wasting my subs. As soon as I put them in, my mouth fills with saliva and drowns the subs in spit. I just let the spit sit there, only swallowing when it becomes to much and making sure not to swallow the film.My question is, are the subs still gonna work properly? Seems like no one has a solution to this problem.	Ive been going to my Suboxone clinic for 4 weeks now. The doctor there said they could also be my primary care physician and has already written me a new script for one of my previous meds, Seroquel. I have been diagnosed with ADHD a long time ago and was taking Adderall/Vyvanse/Ritalin for a few years, but had stopped taking it a while back. Does anyone have any experience getting a script for Adderall from their clinic doc? At least with mine, I know he is a licensed Psychiatrist. Obviously, I know Adderall can be addictive and it could be a bad look trying to ask the doc about it, so just trying to get some advice before I pop the question. Thanks =O

Used to be on buprenorphine	So I posted alot of reports during my jump from 0.5mg. I was on 8mg for about 2.5 years and over the course of 6-8 months I tapered down to 0.5mg. This is what I used for the jump.<br><br>1mg Clonezapam - 2x a day (Morning and Night)<br>0.1mg Clonidine - 2-3x a day<br>300mg Lyrica - 2x a day<br>Immodium (Lopermaid)<br><br>If you go back through my posts you'll see I had a fairly easy time. The helper meds took almost all symptoms away and all I experienced was fatigue. I only took the meds for 2.5 weeks except the Lyrica I ended up taking for almost 6 weeks. My pharmacy and doctor screwed up and refilled it twice before I put a stop to it. It's very addictive and was tough to speak up about it so be careful with it!<br><br>So the update, I wanted to start by saying I think Suboxone is a great way to get off opiates. It helped me emensely. HOWEVER, now that I'm off it I see that it was definately effecting my life negatively and probably should have been stopped sooner then being on it 2.5 years. It's not like I feel completely different now, but I definately feel better. I feel like I'm less in a fog, that I'm more aware of my life and my decisions. I've already managed to lose 10lbs and have been sticking to. Ketogenic Diet that I wasn't ever able to do while on Suboxone. I have way less mood swings and especially don't get very low moods at night anymore. I was waking up every couple hours while on Suboxone and that has now completely dissapeared. Its taken until about 1.5 weeks ago but I can even fall asleep right away and then don't wake up until the morning. I don't have this anxiety about worrying about withdrawal anymore. I just generally feel better. Oh, and I know this doesn't make sense. But I was a chronic relapser while on Suboxone. Only ever going 2-4 months without having a couple days of using opiates. I'm now at 6months without a relapse and I haven't even been getting cravings anymore. Not sure what else to report but feel free to ask me anything. I was also able to stop taking the Proton Pump Inhibitor from horrible gas and bloating I would get while on Suboxone. That's completely been resolved.	Well today is day 42. I am doing it regardless of how hard it seems to be. Still feel like I am moving slow and lots of muscles ache. Stomach is still a little messed up and the bathroom is a lot better. Could use a title advise as to when energy and happiness returns. I am hearing 60 to 90 days. I can dance that. My problem is that no one except my pharmacist even knows that I was on these for 3 years and decided to stop them. I live my my wife and 2 daughters who are fully grown adults. I am over the hard part but need this to get a little better soon. Any advice on this will be helpful. I was a opiate addict for years until all doctors cut me off. Went to sub clinic and off and running what an a hole I was. I am in my 60s so this was not fun at the 2mg jump after doing 16 or a little less for 3 years. I will make this work because I know together we are stronger then Suboxone. Thanks in advance
Cannot discern	Why does it say Do not cut, chew or swallow on the front of the (name brand) Suboxone strips? Don't many people cut the film into smaller doses, and doctors recommend that?	maybe i'm using "irony" wrong? that's the thing to do though, isn't it?<br><br>aaanyway, i was just clicking on sites and such, and i find it.... funny, that a good number of sub docs in my area are actually in boutique practices where they do pain management and physical therapy, ya know, the kinda practices with little water falls and they also sell you special herbs that will "detox" your body? no, not that kind of detox. like purge your intestine of harmful.... whatevers....<br><br>big deal? lots of docs with sub waivers treat pain patients. but no. they do not treat people who have become dependent upon their pain medication. and they don't treat "regular" addicts. they just use the subs as an alternative pain med, which sort of reflects our current panic about addiction.<br><br>get to the point!<br><br>okay, so, what's funny --- these people (the doctors) all spent at least a day of their time in class and got registered with the DEA and waited 45 days to get a new number, had to order new prescription pads (those things add up!) blah blah.....<br><br>you don't need a waiver to prescribe Suboxone for pain. you only need a waiver to prescribe it for addiction.<br><br>of course, if the poor patient doesn't want to spend 3 hours at the pharmacy while the pharmacist and doc exchange voicemails and just wants to get their freaking prescription, it sure helps to have that "X". I just thought it was....<br><br>well i guess my life is pretty uneventful. naw, but i do just like this health policy and following changes in drug laws and all. gave me a kick, thought i'd share it with my online buddies. don't throw stones.
